# Prediction of methane emission from sheep based on data measured *in vivo* from open-circuit respiratory studies

**DOI:** 10.5713/ajas.18.0828

**Published:** 2019-02-07

**Authors:** Tao Ma, Kaidong Deng, Qiyu Diao

**Affiliations:** 1 Feed Research Institute, Chinese Academy of Agricultural Sciences, Key Laboratory of Feed Biotechnology of the Ministry of Agriculture, Beijing 100081, China; 2 College of Animal Science, Jinling Institute of Technology, Nanjing, Jiangsu 210038, China

**Keywords:** Methane, Sheep, Prediction Equation, Digestible Energy, Metabolisable Energy

## Abstract

**Objective:**

The current study analysed the relationships between methane (CH_4_) output from animal and dietary factors.

**Methods:**

The dataset was obtained from 159 Dorper×thin-tailed Han lambs from our seven studies, and CH_4_ production and energy metabolism data were measured *in vivo* by an open-circuit respiratory method. All lambs were confined indoors and fed pelleted diet during the whole experimental period in all studies. Data from two-thirds of lambs were used to develop linear and multiple regressions to describe the relationship between CH_4_ emission and dietary variables, and data from the remaining one third of lambs were used to validate the established models.

**Results:**

CH_4_ emission (g/d) was positively related to dry matter intake (DMI) and gross energy intake (GEI) (p<0.001). CH_4_ energy/GEI was negatively related to metabolizable energy/gross energy and metabolizable energy/digestible energy (p<0.001). Using DMI to predict CH_4_ emission (g/d) resulted in a coefficient of determination (R^2^) of 0.80. Using GEI, digestible energy intake, and metabolizable energy intake predict CH_4_ energy/GEI resulted in a R^2^ of 0.92.

**Conclusion:**

the prediction equations established in the current study are useful to develop appropriate feeding and management strategies to mitigate CH_4_ emissions from sheep.

## INTRODUCTION

There are increasing concerns about the impact of livestock production on the environment. Methane (CH_4_), a greenhouse gas that remains in the atmosphere for approximately 9 to 15 years, is over 25 times more effective in trapping heat in the atmosphere than carbon dioxide (CO_2_) [1]. Livestock farming is a major contributor to atmospheric CH_4_ accumulation. In ruminants, approximately 95.5% of CH_4_ generation is produced by fermentation of feed in the rumen [2], which causes a loss of 2.3% to 10.8% of feed energy depending on the diet and animal [3]. Therefore, national inventories of greenhouse gas emissions are essential for the quantification of these emissions from individual countries and the elaboration of country level mitigation strategies [4]. However, due to the complexity in determining CH_4_ production *in vivo*, prediction equations are essential to accurately estimate CH_4_ emission from ruminants, which is necessary to provide useful strategies for the feeding and management of animals. Although the prediction equation of CH_4_ emission has been established for sheep by different researchers [5–8], it should be noted diet, animal breed, and management system could all contribute to errors when developing national CH_4_ emission inventories [9].

The sheep production in China differs from that in Australia and in Europe, which are almost exclusively dependent upon pasture [10,11]. China is featured by vast population and scarce land per capita, as well as limited forests and pastureland [12]. Since the beginning of the 21st century, the Chinese Government has further implemented polices including “Control grazing for grassland recovery” to conserve grasslands, mitigate degradation, and promote economic development in pastoral regions [13]. On the other hand, China has long history of sheep domestication and currently there are 162.06 million sheep in China, accounting for about 14% of total sheep population in the whole world. Consequently, modern sheep production system in China features limited or even no grazing and the sheep are mainly fed on crop residues. Therefore, it is uncertain if those prediction model based on CH_4_ production from grazing sheep can also accurately predict CH_4_ production from sheep under current feeding conditions in China, which contribute substantially to the world greenhouse emissions due to the large sheep population.

The indigenous breeds such as Hu [14] and thin-tailed Han (small tail Han) [15] sheep are famous for high prolificacy and non-seasonal ovulatory activity. With the introduction of Dorper sheep from Australia in 2001 [16], Dorper sheep×thin-tailed Han crossbred sheep has become a predominant breed for meat-producing in China. In recent years, the nutrient requirements of Dorper×thin-tailed Han crossbred sheep in terms of energy [17–20], protein [21–23], and minerals [24] have been extensively studied and reported. Based on those conditions, dataset used in the current study to provide basic CH_4_ emission parameters were obtained from the same breed (Dorper×thin-tailed Han), feeding conditions (confined indoors), and feeding regime (pelleted diet), which largely mirrors the current characteristics of sheep production system in China. Therefore, in current study, practical equations were established using dataset from our seven previous studies to predict CH_4_ production from Dorper×thin-tailed Han crossbred lambs. Our objective was to provide basic information for the establishment of robust national CH_4_ inventories and practical mitigation strategies to reduce the environmental impact of sheep production systems.

## MATERIALS AND METHODS

### Animals and diets

The dataset used in the present study was obtained from 159 lambs in seven energy metabolism studies undertaken as part of the National Technology Program for the Meat Sheep Industry of China from 2010 to 2015 [17–20,25–27]. The animals were offered pelleted diet in all studies with concentrate:forage ratio ranging from 12:88 to 92:8. The concentrate included corn, soybean, barley, oat, wheat, sorghum, soybean meal, rapeseed meal, cottonseed meal, peanut meal, and dry distillers grains with solubles, and the roughage included Chinese wild rye hay (*Leymus chinensis*) and corn stalk. The data, composed of means, standard deviations and ranges for animal and dietary variables, are presented in Table 1. In each experiment the animals were offered the experimental diets for 4 weeks in group-housed pens before conducting the digestion and respirometry trial to measure energy metabolism. The digestion and respirometry trial comprised a 10-d collection period after a 10-d adaptation period. During the 10-d collection period, feed offered, orts, and faeces were weighed and sampled (10% of total weight) daily. Urine was acidified with 100 mL of 1.8 M H_2_SO_4_ daily and measured for volume, and 1% was sampled daily. As outlined in Deng et al [17], methane production was measured using an open-circuit respirometry system (Sable Systems International, Las Vegas, NV, USA) integrated with 3 metabolism cages each equipped with a polycarbonate head box. On d 0, 2, 4, 6, and 8 of the 10-d collection period, each group of lambs was moved into the metabolism cages for methane assessment. After a 24-h adaptation period, individual methane production was measured over a 24-h period. Methane concentration as well as temperature, humidity, dew point and air flow rate were recorded and processed using the Sable Systems software to calculate individual sheep methane production.

### Statistical analysis

Prediction equations for methane emission were developed using dry matter intake (DMI), neutral detergent fibre intake (NDFI), gross energy intake (GEI), digestible energy intake (DEI), metabolizable energy intake (MEI), dietary metabolisable energy/digestible energy (ME/DE), DE/gross energy (GE) or ME/GE as predictors in multiple regressions. A stepwise multiple regression technique was used to develop multiple prediction equations, and the technique automatically selects the best and significant predictors to fit the prediction equations. Experimental effects on these relationships were removed by the following model:

$$y=a_{i}+b_{1}x_{1}+b_{2}x_{2}+\ldots +b_{n}x_{n},$$

where, *a_i_* represents the effect of experiment *i* for *i* = 1 to 5, *x_1_*, *x_2_*, … *x_n_* are the x-variables and *b_1_*, *b_2_*, … *b_n_* are their regression coefficients. The statistical program used in the present study was Genstat (Version 18.1; VSN International Ltd, Hemel Hempstead, England, 2015).

## RESULTS

### Correlation between methane emission and feed intake as well as energy digestibility and metabolisability

The relationships between CH_4_ and feed intake as well as energy digestibility and metabolisability are shown in Table 2. Total CH_4_ output (L/d) was linearly correlated with feed intake (DMI and NDFI) and energy intake (GEI, DEI, and MEI) (p<0.001). CH_4_ emission expressed as L/kg DMI was linearly correlated with DMI, GEI, DEI (p<0.05), and NDFI (p<0.01). A similar relationship was also observed between CH_4_ energy/GEI and DMI, NDFI, GEI, and DEI. CH_4_ emission expressed as L/kg NDFI was linearly correlated with DEI (p<0.01) and MEI (p<0.05). Total CH_4_ output (L/d), CH_4_ (L/kg DMI), and CH_4_ energy/GEI negatively correlated with ME/GE (p<0.01), while CH_4_ (L/kg NDFI) positively correlated with DE/GE (p<0.01) and ME/GE (p<0.05), and negatively correlated with ME/DE (p<0.05).

### Prediction equations for methane emission and validation of the equations

Using two-thirds of the data, linear regression was established between total CH_4_ emission (L/d) and DMI (g/d) (R^2^ = 0.80) and NDFI (g/d) (R^2^ = 0.76), respectively (Table 3). As the variation in CH_4_ production was best predicted by these two parameters, multiple linear prediction equations were developed using DMI and NDFI (R^2^ = 0.85). Linear regression was established between total CH_4_ energy (MJ/d) and GEI (MJ/d) (R^2^ = 0.80). Multiple regression was established between total CH_4_ energy (MJ/d) and combination of GEI, DEI, and MEI (MJ/d) and the R^2^ of the regression was 0.92. Further validation of those regression models was conducted using the remaining one third of the data (Table 3). The results showed that the average of predicted CH_4_ (L/d) using equations with DMI (38.7), NDFI (41.0), or both DMI and NDFI (40.4) as predicting factors was very close to the actual average of CH_4_ output (30.0). Similarly, the average of predicted CH_4_ energy (MJ/d) using equations with GEI (1.50), and a combination of GEI, DEI, and MEI (1.60) as predicting factors was also close to the actual average of CH_4_ energy (1.57).

### Validation of previously published prediction equations for sheep emissions

The present study used datasets from seven studies (n = 159) to validate previously published prediction equations for CH_4_ emission from sheep (Table 4). The CH_4_ emission (g/d) was under-predicted by Zhao et al [8] but over-predicted by Bell et al [7]. The R^2^ for the relationship between predicted and actual CH_4_ emission (g/d) was close to 0.70. The CH_4_-E was over-predicted using either DMI and GEI by Patra et al [6], or GEI, DEI, or MEI by Zhao et al [8]. The R^2^ for the relationship between predicted and actual CH_4_-E was greatest in Patra et al [6] using DMI (R^2^ = 0.70) or GEI (R^2^ = 0.71) and in Zhao et al [8] using GEI (R^2^ = 0.71), while the lowest R^2^ was observed using MEI as the prediction factor (R^2^ = 0.44). A lower CH_4_/DMI was obtained from the predicted value of Zhao et al [8] and our results (20.8 vs 27.2 g/kg). However, the CH_4_ energy/GEI predicted by Zhao et al [8] was only 71% (5.95/8.37×100) of that measured in the current study. The R^2^ in the relationship between predicted and actual CH_4_/DMI and CH_4_-E/GEI was the 0.62 and 0.59, respectively.

## DISCUSSION

In the current study, average CH_4_ emission was 39.9 L/d or 28.9 g/d, which was comparable to that of Dorper crossbred sheep measured using chambers reported by Nie et al [28] (39.7 L/d) and Zhao et al [8] (37.2 L/d). Furthermore, our result was within the range (12.2 to 37.3 g/d) in studies of grazing sheep summarized by Savian et al [29]. The average CH_4_ scaled to DMI was 37.6 L/kg or 27.2 g/kg in the current study, which was considerably greater than that (16.5 to 21.1 g/kg) reported for sheep fed perennial ryegrass [8,30]. Furthermore, the lower limit of CH_4_ emission scaled to DMI (18.8 L/kg or 13.6 g/kg) in the current study was close to that of Welsh Mountain sheep fed on permanent pasture (14.4 g/kg) or Molinia sheep (14.1 g/kg) [31]. In the current study, pelleted diets were used in all experiments, which theoretically can be more rapidly digested and thus promoting feed intake [32]. A study suggested that pelleting could increase DMI by 45% in sheep, especially for young animals [33], compared with grass. Similarly, we also observed higher DMI (1.04 kg/d) compared with others [8,30,31], which could be attributed to the pelleted diet used in our series of studies. Although it was reported that increasing feed intake can reduce CH_4_ production per unit of feed intake [8], the substantial higher CH_4_ emission (28.9 g/d) compared with others [8,30,31] could be responsible for the higher CH_4_ emission scaled to DMI in the current study. Pinares-Patiño et al [34] reported a lower CH_4_ emission (22.0 g/kg DMI) from ewes also fed pelleted diet measured using chambers. However, it should be noted that the pelleted diet used in their study contained less neutral detergent fibre (NDF, 269 kg/kg DM vs 429 kg/kg DM). Dietary NDF concentration has been proved to be positively correlated with CH_4_ production for ruminants [35]. It was unexpected that CH_4_ emission measured using SF_6_ ranged from 26.7 to 27.9 g/kg DMI for grazing sheep reported by Savian et al [29], which was almost identical to our result. This might be due to the high NDF content in Italian ryegrass (from 586 to 606 kg/kg DM) used in their study. Despite of the dietary factors mentioned above, animal factors (breed, sex, and growth stage) as well as measurement technique can also have influence on CH_4_ emission and therefore should be taken into consideration in the development of mitigation strategies.

CH _4_ emission as a proportion of energy losses accounts for 3.79% to 12.0% of GEI in the current study, which was comparable to the range reported in cattle (2% to 15%) [36]. The average ratio of CH_4_ to total GEI in this study (8.4%) was higher than the average value reported for grazing sheep (6.2%) [8,29,30]. The lower energy utilization efficiency could be again explained by the high passage rate and low nutrient digestibility of sheep fed pelleted diet in the current study. For example, dry matter digestibility (DMD, 61.6%) and organic matter digestibility (OMD, 61.8%) observed in the current study were significantly lower compared with DMD reported by Moorby et al [31] (72.2%) and Zhao et al [8] (73%), and OMD reported by Fraser et al [30] (66.2%). Intergovernmental Panel on Climate Change Tier 2 methodology [37] currently uses GEI along with a standard CH4 conversion factor (CH_4_ energy/GE = 6.5%) to calculate CH_4_ emissions, thus, probably underestimating CH_4_ emission from sheep under the experimental conditions in the current study.

In the current study body weight (BW) was not signifi cantly correlated with CH_4_ emission from sheep. Similarly, it is reported that BW alone is a poor variable for predicting CH_4_ emission in grazing beef cattle (R^2^ = 0.27) [9] and sheep (R^2^ = 0.25) [6], and it was found that metabolic BW was marginally correlated with CH_4_ energy (R^2^ = 0.49) in goats [38], indicating that the accuracy of using BW to predict CH_4_ emission might be affected by feeding conditions.

Feed intake is often used to predict CH _4_ production in inventory models. In the present study, DMI is the main determinant of total CH_4_ emission (R^2^ = 0.80), a result similar to that obtained by Patra et al [6] in sheep (R^2^ = 0.83). It is well documented that CH_4_ emission (L/d) from enteric fermentation in sheep is closely related to total feed intake [8,39]. A strong relationship between DMI and CH_4_ emission was also reported in beef and dairy cattle (R^2^ = 0.68) [40]. A quadratic relationship between CH_4_ energy and DMI in dairy cows was also observed [41]. However, a study suggested that the prediction equations based on DMI as primary predictors of CH_4_ output resulted in a relatively weak R^2^ (0.44) in beef cattle [40]. This might suggest that the inclusion of other variables, such as BW and dietary nutrient concentrations, may be important to improve the predictive accuracy of regression models. Nevertheless, Ellis et al [40] reported that NDFI (kg/d) was the best predictor of CH_4_ production (R^2^ = 0.66) in beef cattle, and further combination of DMI and NDFI could also robustly predict CH_4_ emission from cattle (R^2^ = 0.67), which was in accordance with the regression models established in our study. The NDF fraction contains cell-wall fractions such as cellulose, hemicellulose, and lignin [42]. The positive relationship between NDFI (kg/d) and CH_4_ production in the current study along with the study by Ellis et al [40] might be explained by the dietary NDF concentration, which could improve ruminal fermentation and lead to preferable high acetate:propionate ratio that facilitates CH_4_ production [43], making it an easily measured predictor of CH_4_ production within a regression model.

Energy intake (GEI alone or GEI, DEI and MEI) is also effective prediction factors of CH_4_ emission in the current study, which are in accordance with those observed in cattle [9,40,44]. In agreement with Molano and Clark [45], the quantity of CH_4_ emission, per unit of DMI or GE losses as CH_4_ was not affected by the level of DMI. In the current study, there was a negative relationship between CH_4_/GEI and dietary ME concentrations or ME/DE, which is similar to that reported in beef [9] and dairy cattle [46], indicating that an improved feed utilisation efficiency could reduce CH_4_ emissions. On the other hand, we observed a positive correlation between DMI and CH_4_/GEI, which is inconsistent with previous result in dairy cow [46] and sheep [8]. Indeed, an increase in feeding level (DMI) increases the outflow rate of digesta and thus reduces ruminal nutrient digestion, leading to decrease in CH_4_ [46]. However, in the current study, sheep with higher DMI also consumed relatively more concentrate than those with lower DMI. Previous study suggested that ruminal nutrient digestion increased with increasing concentrate intake [47], which in turn result in the increase in CH_4_ output. Therefore, the positive correlation DMI and CH_4_/GEI observed in the current study can be expected.

Due to the scarcity of relevant studies for sheep, predicted CH_4_ emission parameters using equations from 3 published papers were compared with the actual CH_4_ production in the current study. Both Bell et al [7] and Zhao et al [8] developed prediction models for enteric CH_4_ emissions using sheep in UK, where the sheep production is featured by long grazing seasons [48]. Therefore, the use of those equations in confined-feeding animals must be with caution. Patra et al [6] established prediction model for CH_4_ emission based on the results of more than 1,500 sheep. Although the equations in their study might be more inclusive, it should be noted that the predicting equations established in the current study were more specific in the method (respiratory chamber) used and feeding conditions (confined and fed pelleted diet), which could be more accurate to calculate the CH_4_ inventory under similar conditions.

## CONCLUSION

In the present study, a range of prediction equations for methane production from sheep was based on *in vivo* data from open-circuit respiratory studies. Strong relationships were found between methane production and animal or dietary factors including DMI, NDFI, and GEI. These equations are useful to develop appropriate feeding and management strategies for mitigating methane emission from sheep under current feeding system in China.

## Figures and Tables

**Table 1:** Animal and dietary data (n = 159)

Items	Mean	SD	Minimum	Maximum
Body weight, feed intake and methane data				
BW (kg)	35.4	5.31	23.5	48.9
DMI (kg/d)	1.04	0.27	0.52	2.02
NDFI (kg/d)	0.46	0.16	0.13	0.81
GEI (MJ/d)	18.6	5.04	8.05	36.6
DMD (%)	61.6	7.25	48.2	78.4
OMD (%)	61.8	7.92	46.1	80.2
Methane emission (L/d)	39.9	15.5	14.1	88.9
Methane emission (L/kg DMI)	37.6	6.6	18.8	54.1
Methane energy output (MJ/d)	1.55	0.55	0.56	3.52
Methane energy output/GEI (%)	8.37	1.77	3.79	12.0
Dietary nutrient (kg/kg DM) and energy (MJ/kg DM) concentration				
DM	911.4	18.6	890.0	960.0
CP	135.8	39.0	73.0	236.0
NDF	428.9	125.4	191.0	629.0
GE	16.8	0.7	15.8	18.5
DE	11.2	1.5	4.6	15.6
ME	8.9	1.5	2.2	13.2

SD, standard deviation; BW, bodyweight; DMI, dry matter intake; NDFI, neutral detergent fibre intake; GEI, gross energy intake; DMD, dry matter digestibility; OMD, organic matter digestibility; DM, dry matter; CP, crude protein; NDF, neutral detergent fibre; GE, gross energy; DE, digestible energy; ME, metabolisable energy.

**Table 2:** Significant levels for the linear relationships between methane output from animal and dietary factors

Items	CH_4_ (L/d)	CH_4_ (L/kg DMI)	CH_4_ (L/kg NDFI)	CH_4_ energy/GEI
Feed intake				
DMI (kg/d)	+**	+*		+*
NDFI (kg/d)	+**	+**	−	+**
GEI (MJ/d)	+**	+*		+*
DEI (MJ/d)	+**	+*	+**	+
MEI (MJ/d)	+**		+*	
Energy digestibility and metabolisability				
DE/GE	−	−	+**	−
ME/GE	−**	−**	+*	−**
ME/DE	−**	−**	−*	−**

DMI, dry matter intake; NDFI, neutral detergent fibre intake; GEI, gross energy intake; DEI, digestible energy intake; MEI, metabolisable energy intake; DE, digestible energy; GE, gross energy; ME, metabolisable energy.

‘+/−’ represents 0.1<p<0.05; ‘+*/−*’ represents 0.05<p<0.01; ‘+**/−**’ represents p<0.01.

**Table 3:** Linear and multiple regression for CH4 output using feed intake and energy digestibility and metabolisability

Items	Equation	R^2^	SE	Predicted	Actual
CH_4_ (L/d)	= −5.45(±2.98)+0.043(±0.003)×DMI (g/d)	0.80	9.30	38.7	39.9
CH_4_ (L/d)	= 2.23(±2.76)+0.08(±0.006)×NDFI (g/d)	0.76	9.93	41.0	
CH_4_ (L/d)	= −6.20(±2.74)+0.027(±0.004)×DMI (g/d)+0.039(±0.009)×NDFI (g/d)	0.85	8.54	40.4	
CH_4_ energy (MJ/d)	= −0.19(±0.11)+0.093(±0.006)×GEI (MJ/d)	0.80	0.36	1.50	1.57
CH_4_ energy (MJ/d)	= −0.34(±0.06)+0.043(±0.008)×GEI (MJ/d)+0.65(±0.04)×DEI (MJ/d)−0.70(±0.04)×MEI (MJ/d)	0.92	0.19	1.60	

SE, standard error; DMI, dry matter intake; NDFI, neutral detergent fibre intake; GEI, gross energy intake; DEI, digestible energy intake; MEI, metabolisable energy intake.

**Table 4:** Published equations used for validation of present results

References		Equations	Predicted	Actual	R^2^
Bell et al [7]	CH_4_ (g/d)	= 18+22.5×DMI (kg/d)	41.5	28.2	0.70
Zhao et al [8]		= 3.1+16.7×DMI (kg/d)	20.6		0.70
Patra et al [6]	CH_4_ energy (MJ/d)	= 0.223+0.876×DMI (kg/d)	1.14	1.56	0.70
		= 0.208+0.049×GEI (MJ/d)	1.13		0.71
Zhao et al [8]		= 0.17+0.050×GEI (MJ/d)	1.11		0.71
		= 0.21+0.060×DEI (MJ/d)	0.91		0.58
		= 0.26+0.064×MEI (MJ/d)	0.86		0.44
Zhao et al [8]	CH_4_ (g/kg DMI)	= −2.7+7.9×DE (MJ/kg) − 7.3×ME (MJ/kg)	20.8	27.2	0.62
Zhao et al [8]	CH_4_ (energy/GEI)	= (0.022×DE [MJ/kg] − 0.021×ME [MJ/kg])×100	5.95	8.37	0.59

DMI, dry matter intake; GEI, gross energy intake; DEI, digestible energy intake; MEI, metabolisable energy intake; DE, digestible energy; ME, metabolisable energy.
